# Adipose Tissue Expression of PACAP, VIP, and Their Receptors in Response to Cold Stress

**DOI:** 10.1007/s12031-018-1099-x

**Published:** 2018-07-07

**Authors:** Daemon L. Cline, Landon I. Short, Maeghan A. M. Forster, Sarah L. Gray

**Affiliations:** Northern Medical Program, University of Northern British Columbia, 3333 University Way, Prince George, BC V2N 4Z9 Canada

**Keywords:** PACAP, VIP, Adipose tissues, mRNA expression, Thermogenesis, Lipid metabolism

## Abstract

Obesity arises from disrupted energy balance and is caused by chronically higher energy intake compared to expenditure via basal metabolic rate, exercise, and thermogenesis. The brown adipose tissue (BAT), the primary thermogenic organ, has received considerable attention as a potential therapeutic target due to its ability to burn lipids in the production of heat. Pituitary adenylate cyclase-activating polypeptide (PACAP) has been identified as a key regulator of the physiological stress response both centrally and peripherally. While PACAP has been shown to increase thermogenesis by acting at the hypothalamus to increase sympathetic output to BAT, a peripheral role for PACAP-activated thermogenesis has not been studied. We identified PACAP receptor (PAC1, VPAC1/2) expression for the first time in murine BAT and confirmed their expression in white adipose tissues. PAC1 receptor expression was significantly altered in all three adipose tissues studied in response to 3.5-week cold acclimation, with expression patterns differing by depot type. In primary cell culture, VPAC1 was increased in differentiated compared to non-differentiated brown adipocytes, and the same trend was observed for the PACAP-specific receptor PAC1 in gonadal white fat primary cultures. The primary PAC1R mRNA splice variant in interscapular BAT was determined as isoform 2 by RNA-Seq. These results show that PACAP receptors are present in adipose tissues and may have important functional roles in adipocyte differentiation, lipid metabolism, or adipose sensitization to sympathetic signaling in response to thermogenic stimuli.

## Introduction

Understanding the physiological mechanisms of energy metabolism and thus body weight regulation is critical to reducing prevalence of obesity, which harms billions of people worldwide and imposes unmanageable demands on healthcare systems. Energy homeostasis and the maintenance of healthy body weight is achieved by balancing the amount of energy taken in through food intake with that of energy expended through basal metabolic rate, physical activity and thermogenesis. Remarkably, induction of thermogenesis in brown or beige adipocytes in rodents has been shown to prevent and reverse obesity and improve metabolic homeostasis by enhancing energy expenditure (Cannon and Nedergaard 2004a; Cypess et al. 2009; Lowell and Spiegelman 2000), and thus, the past decade has seen significant interest in targeting thermogenesis as a potential strategy to combat obesity and metabolic disease in humans (Lidell et al. 2014; Ouellet et al. 2011). In order to develop safe therapeutics for obesity, considerable work is required to better understand the cytogenetic pathways regulating the formation of thermogenic adipocytes and the endocrine/autocrine factors that activate them (Kajimura and Saito 2014; Peirce et al. 2014).

Thermogenesis is required to maintain euthermia in the face of changing external temperature and is a physiological process performed predominantly by the adipose tissues (Cannon and Nedergaard 2004b; Cypess et al. 2009; Lowell and Spiegelman 2000; Ouellet et al. 2011). While skeletal muscle also contributes to heat production via shivering thermogenesis (Dubois-Ferriere and Chinet 1981), adipose tissues are required for chronic adaptation to cold via adaptive thermogenesis. Adipose tissue was historically classified as either white adipose tissue (WAT), which is primarily involved in energy storage, or brown adipose tissue (BAT), which is a highly catabolic, thermogenic tissue. The discovery of a third, intermediate, type of adipocytes called brown-in-white “brite”, or “beige” adipocytes, has blurred this definition (Bartelt and Heeren 2014; Harms and Seale 2013; Jespersen et al. 2013; Walden et al. 2012; Wu et al. 2012). These thermogenic cells are distinct in origin from brown adipocytes because their precursors lack MyF5 and arise in select white adipose tissue depots. The exceptional thermogenic capacity of brown and beige adipocytes relies on numerous, densely packed mitochondria containing the inner mitochondrial membrane protein uncoupling protein 1 (UCP1), which uncouples ATP production from mitochondrial respiration, releasing energy in the form of heat (Cannon and Nedergaard 2004b; Enerback et al. 1997; Lowell and Spiegelman 2000; Matthias et al. 2000). The primary stimulus for adaptive thermogenesis is norepinephrine released from sympathetic nerve terminals innervating adipose tissues, which binds the G_s_-protein coupled β3-adrenergic receptors, increasing intracellular cAMP, activation of protein kinase A, and subsequent upregulation of lipolysis and thermogenic gene expression.

The neuropeptide pituitary adenylate cyclase-activating polypeptide (PACAP) is known to be a master regulator of the stress response including the sympathetic response to physiological stress. At the sympathetic adrenomedullary synapse, PACAP is the primary neurotransmitter for the synthesis and sustained release of epinephrine (Eiden et al. 2018) and in other parts of the sympathetic nervous system is expressed in preganglionic neurons. Additionally, PACAP action at the hypothalamus has been shown to induce sympathetic nerve activity (SNA) in target organs, including BAT and WAT (Tanida et al. 2010). PACAP’s role in the central regulation of thermogenesis was first suggested when reserpine-induced hypothermia was reversed with PACAP38 injection into the lateral ventricle of mice in 1995 (Masuo et al. 1995). This was proposed to be mediated by the PACAP-specific receptor PAC1R (Masuo et al. 1995). Temperature-dependent survival in the PACAP-null mouse line was the first genetic evidence to suggest PACAP was critical for the defense of body temperature (Gray et al. 2002). In two independently generated PACAP-null mouse lines, body temperature of PACAP knockout mice was reduced compared to littermate controls following cold exposure (Gray et al. 2002; Tanida et al. 2010), and interscapular BAT (iBAT) collected from PACAP-null pups had lower norepinephrine (NE) as well as its precursor dopamine, whereas circulating epinephrine and NE levels were not reduced (Gray et al. 2002). More recently, it was shown that exogenously administered NE could not induce thermogenic activity of PACAP-null iBAT, as measured by maximal metabolic rate (Diane et al. 2014), suggesting chronic suppression of sympathetic tone in PACAP-null mice due to reduced β_3_ adrenergic receptor expression (Rudecki and Gray 2016).

Pharmacological studies support a role for PACAP in regulating adaptive thermogenesis, as centrally administered PACAP increased body temperature in rat (Pataki et al. 2000), chick (Tachibana et al. 2007), and mice (Banki et al. 2014; Hawke et al. 2009). In contrast, when administered intravenously, PACAP’s thermogenic effect was almost undetectable, suggesting a central mode of action (Banki et al. 2014). More recently, Resch et al. (2013) injected PACAP38 specifically in the ventromedial nucleus of the hypothalamus, which increased body temperature, a concurrent increase in iBAT UCP1 expression and decreased iBAT triglyceride level (Resch et al. 2011; Resch et al. 2013). These effects were not seen when PACAP was injected into the paraventricular nucleus of the hypothalamus (Resch et al. 2013). The central effects of PACAP on thermogenic activity of iBAT has been tied to the SNS through measurement of SNA within the spinal cord and end organs (including iBAT) in response to centrally administered PACAP into the 3rd ventricle (Tanida et al. 2010). Even after C1 surgical spinal transection, a T5/T6 spinal injection of PACAP still increased body temperature and SNA to multiple synaptic beds of mice, although which receptor(s) mediate such effects remain to be elucidated (Inglott et al. 2011). These findings indicate PACAP acts at multiple levels of the brain-thermoeffector circuitry to regulate body temperature in response to cold stress. PACAP mediates its physiological actions by binding to three class B GPCRs (G-protein coupled receptors): PAC1R, VPAC1, and VPAC2 (Harmar et al. 1998). A related neuropeptide, vasoactive intestinal peptide (VIP), binds both VPAC receptors, but not PAC1R. The PACAP-specific receptor PAC1R is one of the most highly spliced GPCRs with 11 known splice variants, and evidence shows that these isoforms are physiologically relevant with respect to modulating target cell response (Chatterjee et al. 1996; Pantaloni et al. 1996; Spengler et al. 1993).

While evidence from the literature suggests PACAP acts centrally or within the autonomic nervous system to regulate thermogenesis, a direct effect of PACAP on thermogenic adipocytes to regulate adipogenesis or adaptive thermogenesis has not been studied. Other neuroendocrine/endocrine factors, such as BMP8b (Whittle et al. 2012) and orexins (Sellayah et al. 2011), that have a central effect on BAT thermogenesis via the SNS, have also been shown to act peripherally at the adipocyte to sensitize thermogenic adipocytes to adrenergic stimulation (Villarroya and Vidal-Puig 2013), and thus, PACAP may also have a peripheral role. Here, we characterize the expression of VIP, PACAP, and the PACAP receptors in three adipose tissue depots of mice and compare the expression levels of these transcripts in the adipose tissues of mice housed at thermoneutrality, when adaptive thermogenesis is not required, to adipose tissues of cold-acclimated mice, when adaptive thermogenesis is fully activated.

## Methods

### Animals

Adult (12-week-old), male wild-type C57BL/6 mice were obtained from Charles River Laboratories (Sherbrooke, QC, Canada). Mice were housed two per cage with sterile corncob bedding and placed on a 12-h light to 12-h dark cycle (lights on 0700–1900 h). Animals had unlimited access to water and standard rodent chow diet (LabDiet 5001, LabDiet, Inc., Brentwood, Leduc, AB, Canada; metabolizable energy 3.02 kcal/g). Mice (*n* = 8/treatment) were housed in thermoneutral (30 °C) (Solace Zone, Alternative design, Siloam Springs, AR) or cold (4 °C) conditions for 3.5 weeks to achieve full cold acclimation (Cannon and Nedergaard 2010). Body weight (g) was measured twice weekly to detect potential declines in mouse health. Care and treatment of mice was in accordance with the guidelines of the Canadian Council on Animal Care, and protocols for the study were approved by the University of Northern British Columbia’s Animal Care and Use Committee.

### Postmortem Analysis

Mice (non-fasted) were sacrificed by cardiac puncture under isoflurane anesthetic, followed by cervical dislocation. Interscapular brown adipose tissue (iBAT), inguinal subcutaneous white adipose tissue (ingWAT), and gonadal (visceral) white adipose tissue (gWAT) were collected by dissection, weighed and flash-frozen in liquid nitrogen, and stored at − 80 °C for RNA analysis or fixed in 10% formalin for histological analysis. These adipose tissue depots were selected as they represent the classic thermogenic brown fat (iBAT), a thermogenic-inducible white fat depot that contains beige adipocytes (ingWAT) and a non-thermogenic intraperitoneal white fat depot (gWAT), thus encompassing the physiologically distinct roles of fat with respect to thermogenesis.

### Time-Domain Nuclear Magnetic Resonance (TD-NMR)

Measurements of body composition (fat, fluid, and lean mass (g)) were collected weekly by TD-NMR using a minispec LF50 (Bruker, Billerica, MA, USA). Unanesthetized mice were placed into an opaque measurement tube, which was inserted into the minispec for a 2-min measurement period.

### Histological Analysis of Adipose Tissue

The stored lipid content of brown adipose tissue is reduced during adaptive thermogenesis as part of cold acclimation (Bukowiecki et al. 1982; Picard et al. 2002). As a representative measure of lipid content, the mean area of lipid droplets within the adipose tissue sections was measured using CellSens software (Olympus, Richmond Hill, ON, Canada) with an Olympus BX61 microscope and attached Olympus DP72 camera. Adipose tissues were fixed in 10% formalin for 36 h and then stored in 70% ethanol until analysis. Samples were embedded in paraffin, sectioned (5 μm) and stained with hematoxylin and eosin (H&E) (Wax-it Histological Services, Vancouver, BC, Canada). Ten regions of interest (ROI) were examined across four slides for each mouse (*n* = 4). Photographs of the ROIs were taken, contrast was digitally maximized and the images binarized to black and white. Non-lipid area was calculated by CellSens software as black areas in the ROI, and this area was then subtracted from the total area to give the lipid area, which was then divided by total area to give lipid area as a percentage of the total area. The number of nuclei present per ROI was counted manually to determine the number of cells per ROI. To mitigate bias, all photographs and calculations were performed by the same individual who was blinded to the treatment of each sample.

ingWAT becomes heterogeneous with cold acclimation, including both white and beige adipocytes as the requirement for adaptive thermogenesis increases, whereas gWAT undergoes minimal browning with cold exposure. Histological sections of ingWAT and gWAT were prepared as above and photographed.

### Adipose Tissue Fractionation

PACAP and PAC1R expression are known to occur in immune cells, such as macrophages, that have been isolated from adipose tissues, and therefore, tissues were fractionated and expression of PACAP-related genes measured in the stromal vascular and mature adipocyte fraction. Non-fasted, male C57BL/6 mice (*n* = 6) were sacrificed by cardiac puncture under isoflurane anesthetic, followed by cervical dislocation. Fat depots were dissected and trimmed to remove contaminating material: muscle and white fat from the iBAT, lymph nodes from the ingWAT, and reproductive tissues from the gWAT. Adipose tissues from six mice were pooled by type and minced into 1–2-mm pieces and digested in 10 mL Hank’s Balanced Salt Solution with 2% Bovine Serum Albumin (Sigma Aldrich, St. Louis, MO, USA), 150 U/mL Type II Collagenase (Sigma Aldrich), and 5 mM CaCl_2_ at 37 °C, at 200 rpm in a two-directional shaker for 1 h. Tubes were inverted every 15 min to aid in dissociation of cells. Tissue slurries were strained (300 μm) and fractionated by centrifugation at 1000 rpm for 5 min at 4 °C. Mature adipocytes were collected carefully from the floating layer, supernatant was discarded, and the pelleted stromal-vascular fraction (SVF) was collected. The collected fractions were flash-frozen in liquid nitrogen and stored at − 80 °C until RNA extraction.

### Primary Tissue Culture

To assess PACAP receptor expression in pre-adipocytes or mature adipocytes without contaminating vasculature, neural innervation or immune cells, adipocytes were grown in primary culture, and either differentiated or not. Fourteen-week-old, male C57BL/6 mice (*n* = 24) were sacrificed and adipose tissues collected by dissection. Digestions were completed for each tissue as above; then, debris and undigested material were removed using a tissue strainer (100 μm) and red blood cells removed using Red Cell Lysis Buffer (Sigma Aldrich). The cells were washed by centrifugation and resuspension in DMEM/F12 three times, then plated and cultured at 37 °C, 5% CO_2_ in 6 wells of a 6-well plate until confluent. Growth media contained DMEMF12, 0.01 M HEPES buffer, 10% FBS, and 1% *v*/*v* antibiotic (penicillin/streptomycin) (Sigma Aldrich). One day post-confluence, half the wells for each tissue type were collected for RNA extraction (*n* = 3) and the other half (*n* = 3) were differentiated in media containing: 1.5 nM triiodothyroxine, 4 nM insulin, and 25 μg/mL sodium L-ascorbate for 6 days. Full differentiation was assessed in primary adipocytes by the presence of large lipid droplets in 70% of cells. For primary brown adipocyte cultures, maturation was confirmed post hoc by measuring induction of UCP1 mRNA.

### RNA Extraction and cDNA Generation

All equipment and reagents were acquired from Thermo Fisher Scientific (Waltham, MA, USA) unless otherwise stated. Samples of iBAT, ingWAT, and gWAT were homogenized completely using a PowerGen 125 power homogenizer according to tissue and treatment type. Cells were lysed and RNA extracted and purified using Trizol Reagent and subsequent ethanol precipitation as per the manufacturer’s protocol, with the following modifications: the extra centrifugation step for high fat samples was utilized and RNA precipitation was conducted for 1 h at − 20 °C, then 10 min at − 80 °C. Purity was assessed by spectrophotometry (NanoDrop 1000). Integrity of the RNA was assessed by the quality of 18S and 28S bands on a native 1.5% agarose gel stained with 0.5% ethidium bromide. DNA contamination was removed by treating extracted RNA with TURBO-DNase as per the manufacturer’s protocol. RNA was reverse transcribed to cDNA using iScript Reverse Transcription Supermix for RT-qPCR (Bio-Rad; Hercules, CA, USA) as per the manufacturer’s protocol. Additionally, no-template and no-RT negative controls were performed to test for contaminating genomic DNA.

RNA extractions for samples from primary cell culture and fat fractionation experiments were performed using RLT lysis buffer and RNeasy Mini and RNeasy Micro kits, respectively (QIAGEN, Hilden, Germany). Furthermore, RNA integrity from fat fractions was assessed using the Experion (Bio-Rad) automated electrophoresis system using RNA StdSens kits according to the manufacturer’s instructions.

### Real-Time Quantitative PCR (qPCR)

Melt curves and amplification efficiency were determined for all primers (Integrated DNA technologies (IDT), Coralville, CA, USA) to test for presence of nonspecific products and primer-dimer formation, respectively (Table 1). Homogeneity of reference gene expression across treatments was determined (geNorm, qBase ^+^ software, Biogazelle, Zwijnaarde, Belgium). Primers and hydrolysis probes (for PACAP and VPAC2 only, PrimeTime probes, IDT) were designed using the NCBI PrimerBLAST tool. SYBR Green chemistry (SYBR Green Supermix for iQ5, Bio-Rad) was used to measure mRNA expression of uncoupling protein 1 (UCP1), β-3 adrenergic receptor (ADRB3), homeobox protein Hox-C9 (HOXC9), hormone-sensitive lipase (HSL), PAC1R, VPAC1, and VIP. Amplification conditions were as follows: 95 °C/3:00 min, 95 °C/10 s then (annealing T)/30 s (repeat 40 times) (see Table 1 for annealing temperatures). Melt curve was performed by 55 °C/10 s + 0.5 °C per repeat up to 95 °C. mRNA expression for each gene was analyzed using the comparative C_T_ method.

**Table 1:** Primer and probe sequences used in real-time quantitative PCR

Target Genes	Forward primer (5′-3′)	Reverse primer (5′-3′)	Annealing (**°**C)
UCP1	CCTGGCAGATATCATCAC	TCACCTTGGATCTGAAGG	52
HSL	GGAGCACTACAAACGCAACGA	TCGGCCACCGGTAAAGAG	55
ADRB3	CAACCCGGTCATCTACTG	ACCGTAGCTACACAGAAG	50
HOXC9	GCAGCAAGCACAAAGAGGA	CGTCTGGTACTTGGTGTAGGG	58
PACAP	ATCCAGCGGACAGGAGAGAT	TCCGAGTGGCGTTTGGTAAG	55
PAC1R	TTGATGACTATGAGCCCGAGT	ACAAGATGACCATGGCAGTG	57
VIP	CAGGAACCGGGAACAGACT	TATCAGGAATGCCAGGAACT	57
VPAC1	AAGTCATTGTAGAGGCAGAT	AATATGTCAAGACGGAATCAG	59
VPAC2	AGAGCCATCTCTGTGCTGGTCAA	AGGTAGGCCAGGAAGCAC	57
Hydrolysis probes (5′-3′)			
VPAC2	56FAM/CAGGTAGAG/ZEN/ACCCTCCACCAGAAGCCAGTAG/3IABkFQ		
PACAP	56FAM/TCGCCCACG/ZEN/AAATCCTTAACGAAGCCTATCGAA/3IABkFQ		
Reference genes	Forward primer (5′-3′)	Reverse primer (5′-3′)	
β-actin	GCTCTGGCTCCTAGCACCAT	GCCACCGATCCACACAGAGT	55
GAPDH	TGCACCACCAACTGCTTAG	GGATGCAGGGATGATGTTC	55
RPL19	CCATGAGTATGCTCAGGCTACAG	CTGATCTGCTGACGGGAGTTG	57.5
TBP	CACCAATGACTCCTATGA	CCAAGATTCACGGTAGATA	53
18S	CGGCTACCACATCCAAGGAA	GCTGGAATTACCGCGGCT	55

MIQE guidelines were adhered to in the design and optimization of primers and probes, as well as the determination of relative expression for all of the above genes. UCP1 mRNA is known to be upregulated in brown and beige adipocytes during cold acclimation. ADRB3 and HSL are also induced in adaptive thermogenesis. HOXC9 is expressed in white and beige adipocytes. Expression analysis of these genes was included to assess the relative induction of adaptive thermogenesis in the adipose tissue depots in response to cold acclimation.

### RNA Sequencing

To determine which PAC1R splice variants were most prevalent in iBAT samples of thermoneutral and cold-acclimated mice, mRNA (1 μg) was sequenced at a depth of 25 million paired-end reads on a NextSeq 500 sequencing system (Illumina, San Diego, CA, USA) at the Biomedical Research Centre (UBC, Vancouver, BC, Canada). Samples (*n* = 3/group) were chosen based on RNA-Quality Index (RQI) values obtained from Experion electrophoresis following TURBO DNase treatment. Libraries were prepared using the TruSeq Stranded mRNA Library Prep Kit and analysis of results was performed in the BaseSpace Sequence Hub (Illumina).

### Statistical Analysis

All quantitative data were analyzed by multiple *t* tests (*α* = .05 for each time interval) to determine significance between the two treatment groups.

## Results

### In Vivo Model of Cold Acclimation for Analysis of PACAP, VIP, and PACAP Receptor Expression

#### Body mass and composition

**Fig. 1:**
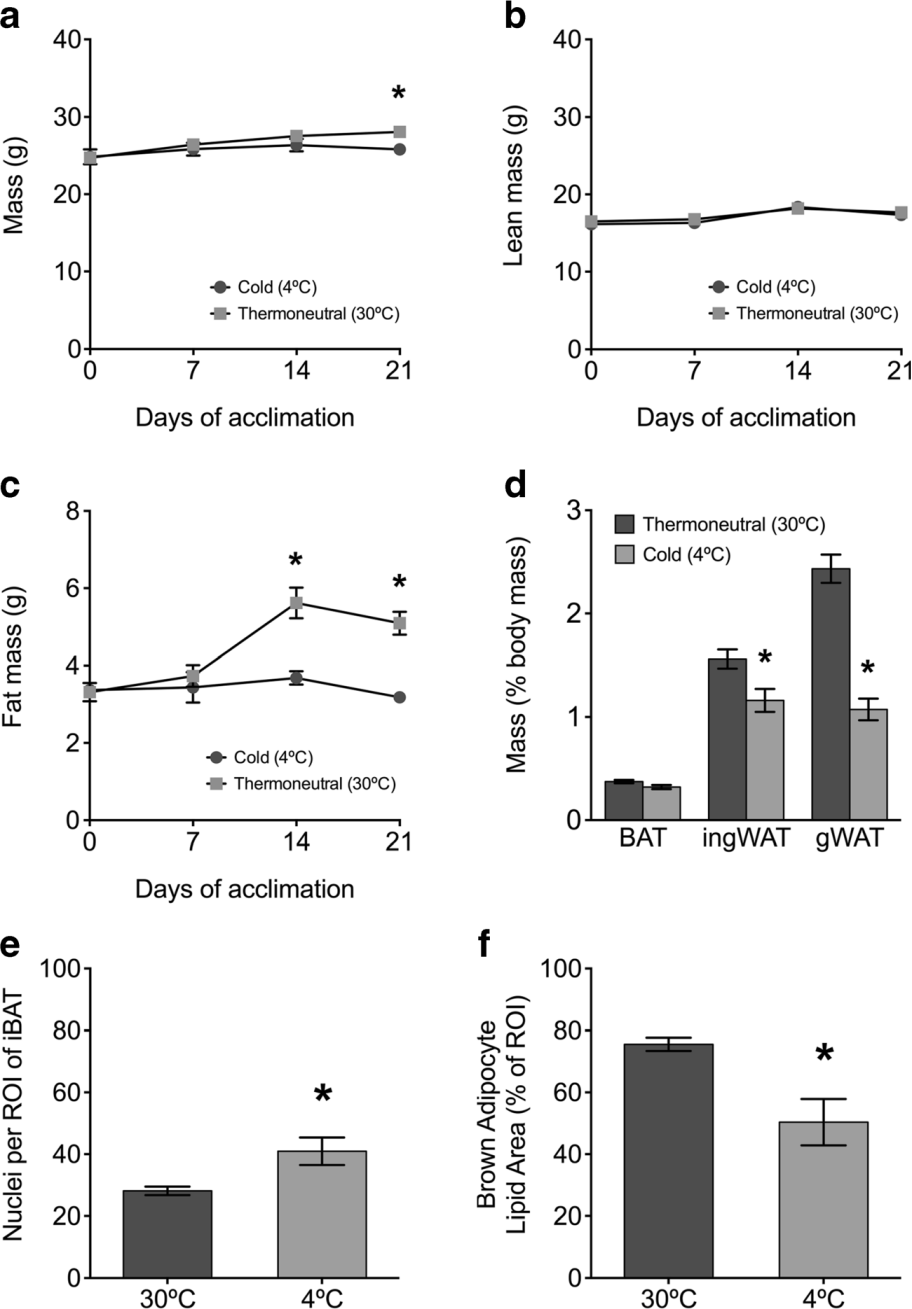
Body composition and morphological assessment of adipose tissues in a 3.5-week cold-acclimation model compared to mice housed at thermoneutrality. Data are expressed as mean ± S.E.M. unless not detected (n.d.), and asterisks denote a significant temperature effect at *α* = 0.05 (30 °C vs. 4 °C). **a** Body mass, **b** lean mass, and **c** fat mass were measured by time-domain nuclear magnetic resonance (Bruker minispec LF50) (*n* = 8 for days 0 and 21, *n* = 4 for days 7 and 14). Sample size differs due to technical problems. **d** Adipose tissue mass (% body mass) in acclimated mice (*n* = 8). Histological analysis of iBAT from acclimated mice show **e** lipid area as a percentage of total region of interest (ROI) area and **f** number of nuclei per ROI (*n* = 4)

At the beginning of the study (d0), body mass (g), lean mass (g), or fat mass (g) did not differ between mice assigned to the two groups (thermoneutral and cold housing). After 3 weeks, cold-acclimated mice had significantly lower body mass than mice housed at thermoneutrality for the same period (Fig. 1a). This was associated with significantly lower fat mass in the cold-acclimated mice after two weeks compared to mice housed at thermoneutrality with no significant difference in lean mass (Fig. 1b, c).

#### Adipose tissue mass

gWAT and ingWAT masses were significantly reduced in cold-acclimated mice compared to mice housed at thermoneutrality, but iBAT mass did not differ between treatments (Fig. 1d).

#### Morphological analysis of adipose tissue

**Fig. 2:**
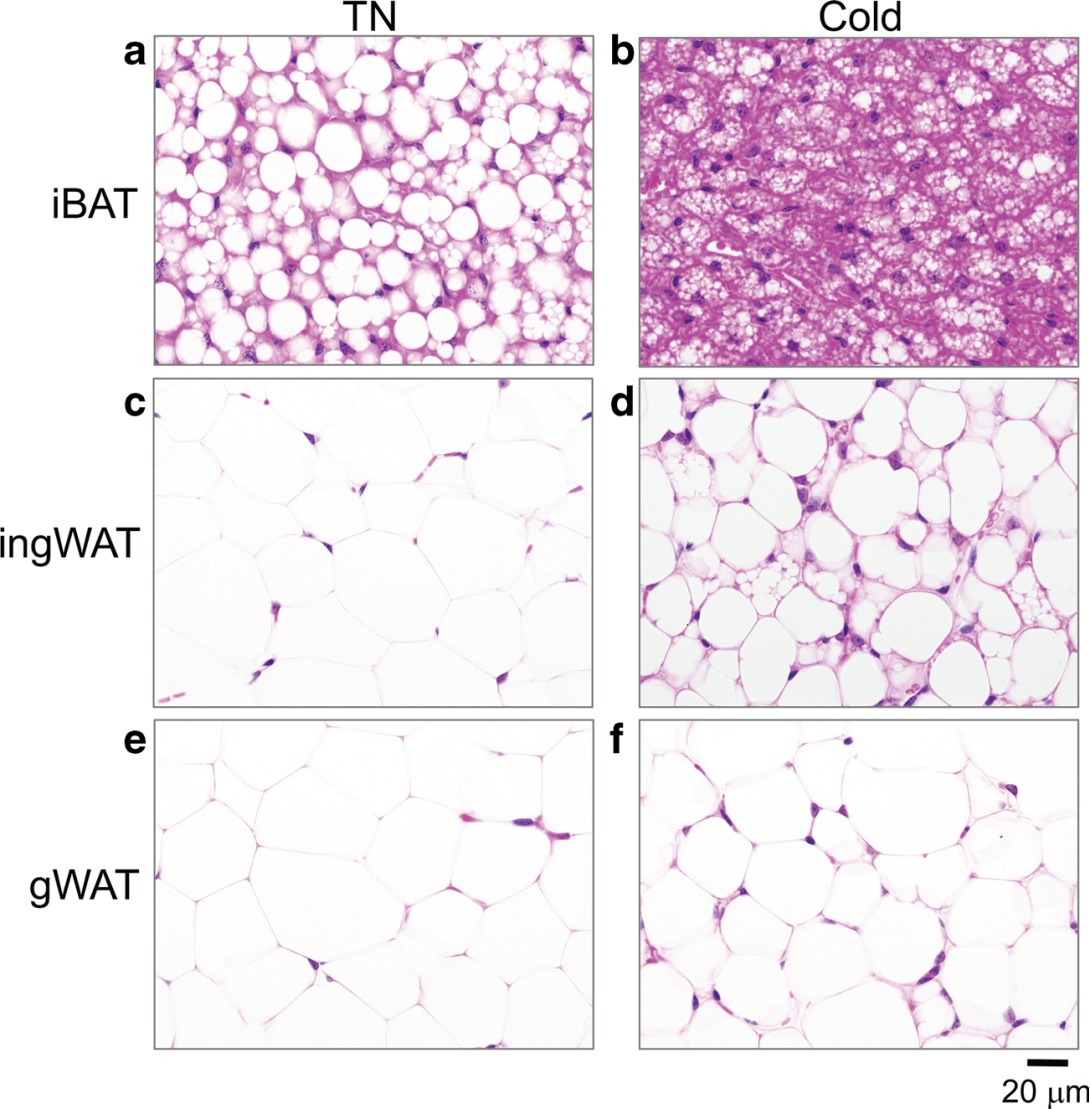
**a**–**f** Representative bright field images of H+E stained sections of interscapular brown adipose tissue (iBAT), inguinal white adipose tissue (ingWAT) and gonadal white adipose tissue (gWAT) from mice acclimated to cold (4 °C) compared to thermoneutrality (30 °C, TN). All images were taken at × 600 magnification

The area of lipid within iBAT sections was significantly reduced in the cold-acclimated mice compared to the thermoneutral group (Fig. 1e). Additionally, the mean number of cells per ROI was significantly greater in the cold-acclimated mice, suggesting smaller adipocytes and supporting the findings of reduced lipid content/cell (Figs. 1 f and 2a, b). The cold-acclimated ingWAT was more heterogeneous than ingWAT adipose from the thermoneutral group, with increased prevalence of multilocular adipocytes characteristic of thermogenic beige adipocytes (Fig. 2c, d).

#### Molecular analysis of adipose tissue

**Fig. 3:**
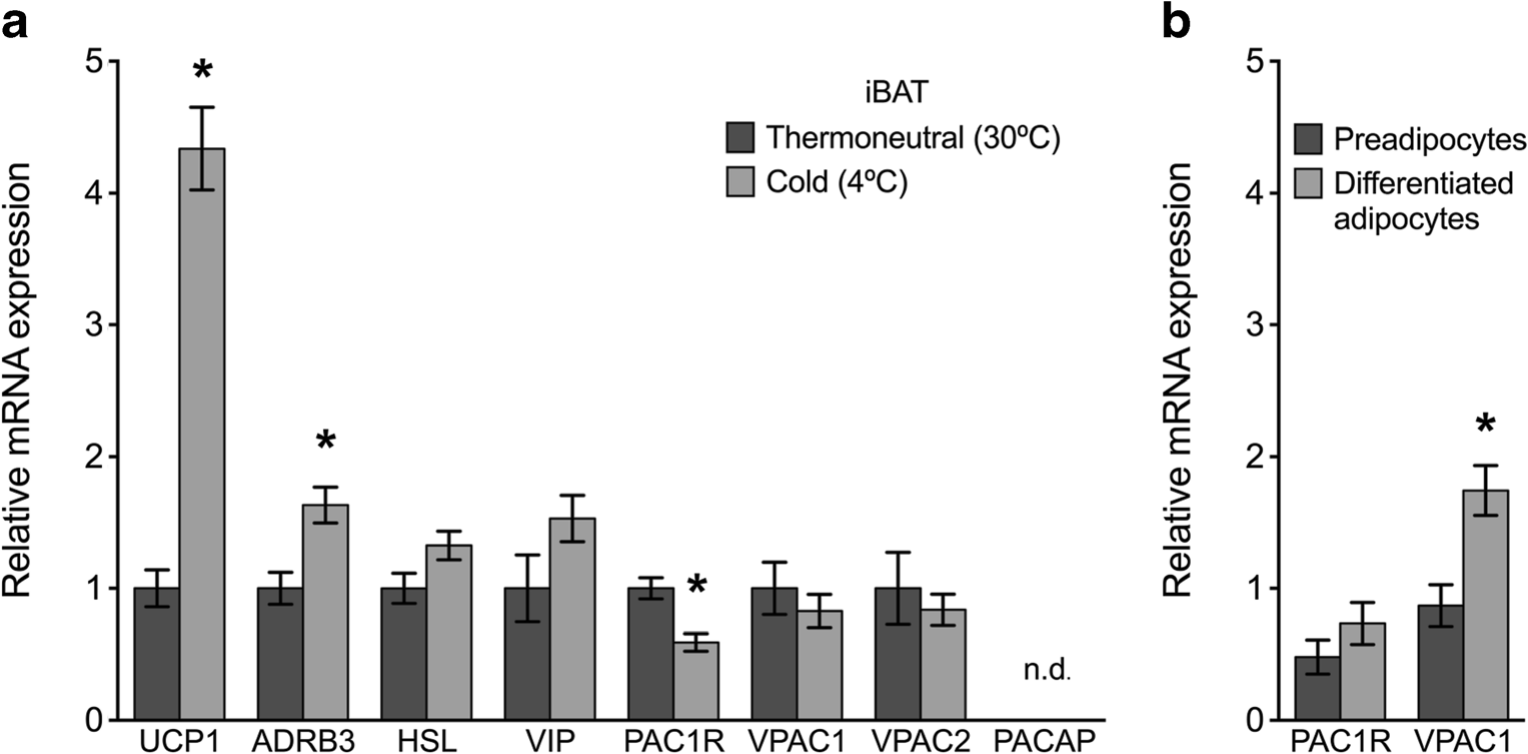
**a** Relative mRNA expression of target genes in interscapular brown adipose tissue (iBAT) of mice acclimated to thermoneutrality (30 °C) or cold (4 °C) for 3.5 weeks (*n* = 8/group). Expression was normalized to reference genes TBP and RPL19. Data are expressed as mean ± S.E.M. unless not detected (n.d.) and asterisks denote a significant temperature effect at *α* = 0.05 (30 °C vs. 4 °C). **(b)** Relative mRNA expression of PACAP receptors in adipocyte primary cultures from iBAT of non-acclimated mice (*n* = 3/group). Expression is normalized to the reference gene RPL19. Data are expressed as mean ± S.E.M., and asterisks denote a significant effect of differentiation at *α* = 0.05 (preadipocytes vs. differentiated, mature adipocytes)

**Fig. 4:**
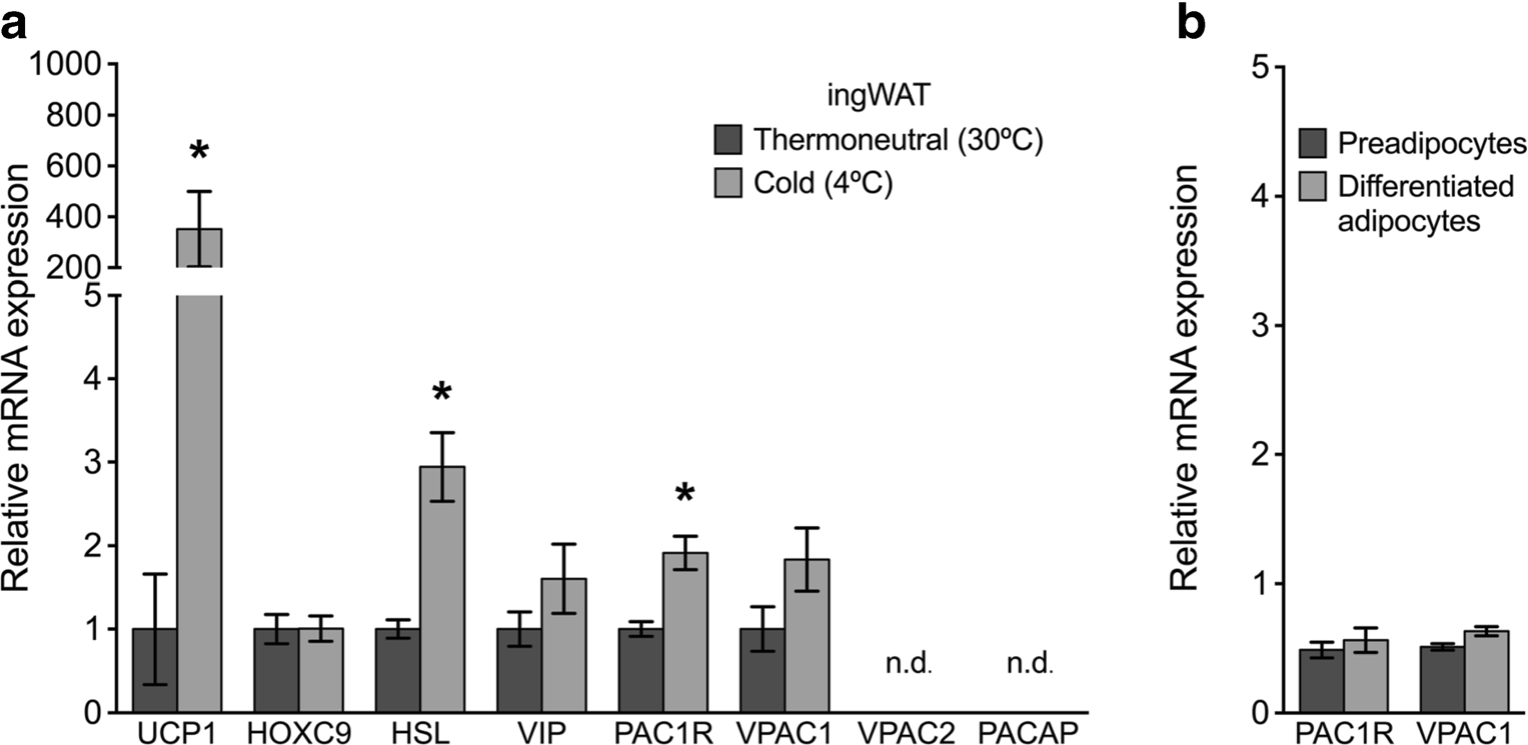
**a** Relative mRNA expression of target genes in inguinal white adipose tissue (ingWAT) of mice acclimated to thermoneutrality (30 °C) or cold (4 °C) for 3.5 weeks (*n* = 8/group). Expression was normalized to reference genes 18S, β-actin, and GAPDH. Data are expressed as mean ± S.E.M. unless not detected (n.d.), and asterisks denote a significant temperature effect at *α* = 0.05 (30 °C vs. 4 °C). **b** Relative mRNA expression of PACAP receptors in adipocyte primary cultures from ingWAT of non-acclimated mice (*n* = 3/group). Expression is normalized to the reference gene RPL19. Data are expressed as mean ± S.E.M., and asterisks denote a significant effect of differentiation at *α* = 0.05 (preadipocytes vs differentiated, mature adipocytes)

**Fig. 5:**
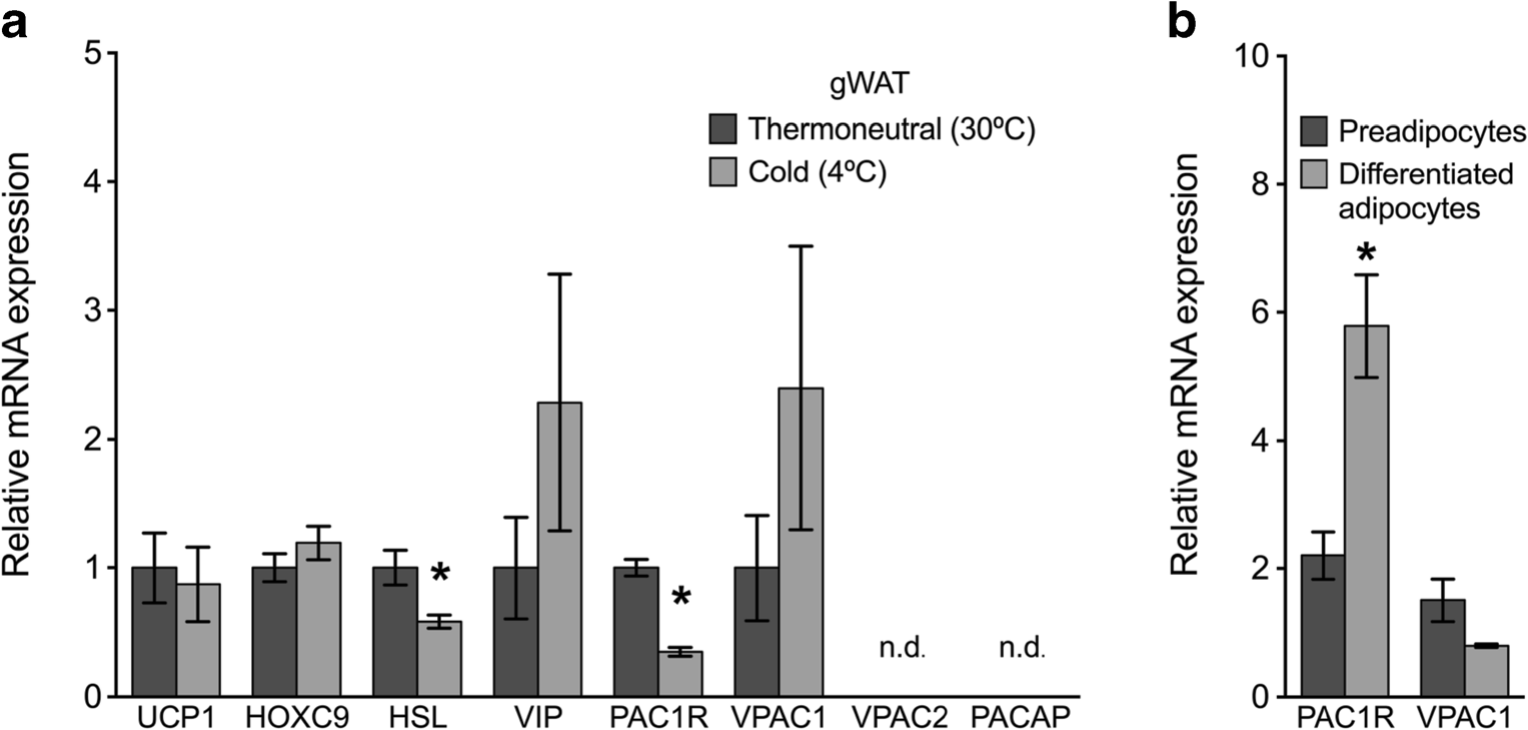
**a** Relative mRNA expression of target genes in gonadal white adipose tissue (gWAT) of mice acclimated to thermoneutrality (30 °C) or cold (4 °C) for 3.5 weeks (*n* = 8/group). Expression was normalized to reference genes β-Actin, RPL19, and TBP. Data are expressed as mean ± S.E.M. unless not detected (n.d.), and asterisks denote a significant temperature effect at *α* = 0.05 (30 °C vs. 4 °C). **b** Relative mRNA expression of PACAP receptors in adipocyte primary cultures from gWAT of non-acclimated mice (*n* = 3/group). Expression is normalized to the reference gene RPL19. Data are expressed as mean ± S.E.M., and asterisks denote a significant effect of differentiation at *α* = 0.05 (preadipocytes vs differentiated, mature adipocytes)

As expected, mRNA for the thermogenic protein UCP1 and β-3 adrenergic receptor (ADRB3) was significantly upregulated in the cold-acclimated iBAT compared to thermoneutral iBAT (Fig. 3a). mRNA expression for HSL was not significantly upregulated in cold acclimated iBAT samples (Fig. 3a). UCP1 mRNA was also significantly upregulated in cold-acclimated ingWAT samples compared to thermoneutral ingWAT (Fig. 4a). In ingWAT, HSL mRNA was significantly increased with cold-acclimation. As expected, UCP1 expression was not regulated in response to housing temperature in gWAT samples, a non-thermogenic white adipose tissue depot (Fig. 5a). HSL mRNA was significantly downregulated in cold-acclimated gWAT samples compared to gWAT samples from mice housed at thermoneutrality. Gene expression of HOXC9, a protein expressed in both white and beige adipocytes, was not significantly different in ingWAT or gWAT of the treatment groups (Figs. 4 a and 5a).

##### Brown Adipose Tissue Expresses VIP and PACAP Receptors, and PAC1R Expression Is Downregulated with Cold Acclimation

The PACAP receptors, PAC1R (23.0 ± 0.838C_T_), VPAC1 (33.2 ± 0.232C_T_), and VPAC2 (32.5 ± 0.744C_T_), were expressed in iBAT samples from cold-acclimated and thermoneutral-housed mice (Fig. 3a). In contrast, PACAP mRNA was not detectable by qPCR in iBAT samples (limit of detection > 35C_T_) from either treatment group, despite being detected in a 300-fold serial dilution of hypothalamus cDNA using our reaction conditions (not shown). VIP was detected in iBAT samples from both treatment groups (32.3 ± 0.213C_T_). Of the receptors expressed in iBAT, PAC1R was the only receptor whose expression was differentially expressed with housing temperature, being significantly downregulated in iBAT samples from cold-acclimated mice compared to thermoneutral mice (Fig. 3a). RNA sequencing confirmed the above results and showed that the predominant PAC1R mRNA splice variant in iBAT was isoform 2 (accession: NM_001025372.2). The NCBI tool COBALT was used to align sequences and determine the presence of a full extracellular domain sequence, but lack of the HIP or HOP cassettes in the 3rd intracellular loop domain of the PAC1R.

Review of qPCR melt curves revealed PAC1 and VPAC1 receptors to be expressed in both the SVF and mature adipocyte fraction of iBAT samples. Because pooled samples were used for this experiment, standard error could not be reported, and thus, we have not commented on the level of expression in these samples. VPAC1 mRNA expression was significantly increased in mature, differentiated adipocytes compared to undifferentiated adipocytes in primary cell cultures derived from brown adipose tissue (Fig. 3b). PAC1R expression also demonstrated a trend of increased expression in differentiated vs undifferentiated adipocytes; however, this result was not statistically significant (Fig. 3b).

##### Inguinal White Adipose Tissue Expresses VIP and PACAP Receptors, and PAC1R Expression Is Upregulated with Cold Acclimation

Two of the three PACAP receptors, PAC1R (33.8 ± 0.1027C_T_) and VPAC1 (31.8 ± 0.187C_T_), were expressed in ingWAT samples from both cold-acclimated and thermoneutrally housed mice (Fig. 4a). In contrast, VPAC2 and PACAP mRNA were detected in only a few ingWAT samples and in all samples exceeded or approached the limit of detection (> 35C_T_). VIP mRNA was detected in ingWAT samples from both treatment groups (32.00 ± 0.166C_T_). PAC1R mRNA was significantly upregulated in ingWAT samples from cold-acclimated mice compared to thermoneutral mice (Fig. 4a).

Expression of PAC1R and VPAC1 mRNA were confirmed in fractionated samples in both the SVF and the mature adipocyte layer by reviewing qPCR melt curves. Because pooled samples were used for this experiment, standard error could not be reported and thus we have not commented on the level of relative expression between groups. In primary cultures of ingWAT-derived adipocytes, PAC1R and VPAC1 mRNA expression did not differ significantly between differentiated and non-differentiated cells (PAC1R *p* = 0.54 and VPAC1 *p* = 0.09) (Fig. 4b).

##### Gonadal White Adipose Tissue Expresses VIP and PACAP Receptors, and PAC1R Expression Is Upregulated with Cold Acclimation

Two of the three PACAP receptors, PAC1R (31.8 ± 0.275C_T_) and VPAC1 (29.7 ± 0.229C_T_) were expressed in gWAT samples from both cold-acclimated and thermoneutrally housed mice (Fig. 5a). In contrast, VPAC2 receptor mRNA and PACAP mRNA were detected in only a few gWAT samples with all samples revealing expression levels at or approaching the limit of detection (> 35C_T_). VIP was detected in ingWAT samples from both treatment groups (31.7 ± 0.276C_T_). We observed variable expression of VIP and the VPAC1 receptor in cold-acclimated gWAT samples, and thus, no significant difference between treatment groups was identified. PAC1R mRNA expression was downregulated in cold-acclimated gWAT samples compared to gWAT samples from thermoneutral mice (Fig. 5a).

Review of qPCR melt curves revealed PAC1R and VPAC1 expression in both SVF and mature adipocyte fractions isolated from gWAT samples. Because pooled samples were used for this experiment, standard error could not be reported, and thus, we have not commented on the level of relative expression between groups. In primary cultures of gWAT-derived adipocytes, PAC1R was more highly expressed in differentiated cells than preadipocytes (Fig. 5b).

## Discussion

PACAP has been shown to regulate the thermogenic response to cold stress (Diane et al. 2014; Gray et al. 2002) via activation of the sympathetic nervous system (Tanida et al. 2010). Evidence suggests this regulation occurs at the level of the hypothalamus (Resch et al. 2011; Resch et al. 2013; Tanida et al. 2010) and/or via regulation of norephinephrine secretion from postganglionic nerves (May and Braas 1995; Rudecki and Gray 2016). However, it is yet unclear whether PACAP is an important neurotransmitter in the pre- or post-ganglionic synapse in nerve terminals of the sympathetic nervous system innervating adipose tissues, and a direct role for PACAP in regulating thermogenic adipocyte function has not been investigated. As a first step, we analyzed the expression of VIP, PACAP, and the PACAP receptors in the thermogenic adipose tissue iBAT, in thermogenically inducible ingWAT and in non-thermogenic gWAT. The PACAP-specific receptor, PAC1R has previously been shown to be expressed in visceral white adipose tissues from humans (Yang et al. 2003), rat adipose tissue collected near pancreas and blood vessels, and 3T3-L1 cells (Nakata et al. 1999). VPAC2 expression and effects on lipolysis have been discussed in cultured primary adipocytes from gWAT in rat (Akesson et al. 2005; Akesson et al. 2003). However, detailed characterization of PACAP, VIP, and PACAP receptor expression in a brown adipose tissue, thermogenic-inducible adipose tissue, or in brown and white adipose tissues in response to environmental temperature has, to our knowledge, not previously been investigated.

All adipose tissue depots analyzed expressed one or more PACAP receptor subtypes. In iBAT, PAC1R was most highly expressed and VPAC1 and VPAC2 were also detected (Fig. 3a). Sequencing of iBAT RNA further revealed the prevalent splice variant of the PAC1R to be isoform 2, which includes the full-length extracellular domain, but lacks both the HIP and HOP cassettes in the third intracellular loop (accession: NM_001025372.2). The absence of the HIP cassette suggests that this variant has potent adenylate cyclase and phospholipase C activating properties (Spengler et al. 1993). Since PACAP and PACAP receptors are implicated in the circulatory system (Otto et al. 2004; Seeliger et al. 2010), and brown adipose tissue is highly vascularized, we confirmed receptor expression in adipocytes themselves. We first showed PAC1R and VPAC1 mRNA to be detected in both the SVF and mature adipocyte fraction of iBAT. Furthermore, PAC1R and VPAC1 were expressed in primary preadipocyte and differentiated adipocyte cultures derived from iBAT (Fig. 3b). Taken together, these data indicate PAC1R mRNA expression occurs by adipocytes themselves. VPAC1 expression was significantly higher in primary brown adipocytes after differentiation, and though not significant, PAC1R expression had the same trend. These results suggest that VPAC1 (and possibly PAC1R) may be important in the maturation of BAT-derived preadipocytes to thermogenically competent, mature adipocytes.

In both ingWAT and gWAT, PAC1R and VPAC1 were expressed in the whole tissue transcriptome (Figs. 4 a and 5a) and were expressed in the SVF, mature adipocyte fraction, and cultured primary pre-adipocytes and differentiated adipocytes (Figs. 4 b and 5b). PAC1R expression was significantly increased in differentiated adipocytes derived from gWAT compared to preadipocytes, suggesting PACAP may play an important functional role in maturation of non-thermogenic adipocytes. Surprisingly, VPAC2 was not detectable in either of the white fat depots despite previous work demonstrating a functional effect of VPAC2 in the activation in lipolysis and attenuation of insulin-mediated glucose uptake in cultured primary adipocytes and 3T3-L1 cells (Akesson et al. 2005; Akesson et al. 2003; Nakata et al. 1999).

PACAP itself was not expressed in any of the three adipose tissues suggesting PACAP is not an adipokine, and instead acts in a paracrine fashion once released from post-ganglionic sympathetic nerves innervating adipose tissues, or as a classical endocrine factor reaching adipocytes via the capillary network feeding adipose tissue. In contrast, VIP was detected in all adipose tissue depots (Figs. 3, 4, and 5). VIP is known to be expressed in the nerves innervating smooth muscle, including peripheral blood vessels (Ivic et al. 2017; Lundberg et al. 1984; Uddman et al. 1981), and thus, VIP mRNA detection may not be representative of expression in adipocytes.

Given PACAP receptor expression in thermogenic adipose tissue was verified, we utilized a well-established model of cold acclimation to determine if VIP, PACAP, and its receptors were differentially expressed in adipose tissues in response to environmental temperature, comparing mice housed at 4 °C for 3.5 weeks (cold-acclimated) to mice housed at 30 °C (thermoneutrality) (Walden et al. 2012). Decreased body weight due to decreased fat mass was observed in cold-acclimated animals due to an increase in the mobilization of lipids to fuel adaptive thermogenesis (Fig. 1). This was reflected in significantly reduced ingWAT and gWAT depots (Fig. 1d) with smaller white adipocytes in the cold-acclimated mice compared to the thermoneutral group (Fig. 2c–f). iBAT of cold-acclimated mice had significantly reduced cellular lipid content and increased cell density compared to iBAT from thermoneutral mice, a phenotype characteristic of activated brown adipose tissue (Figs. 1 e, f and 2a, b).

At the molecular level, expression of the thermogenic protein UCP1 was significantly increased with cold exposure in thermogenic adipose tissues (iBAT and ingWAT) but not gWAT. Increased sympathetic activity of cold-acclimated iBAT was reflected by increased ADRB3 expression (Fig. 3a). Hormone-sensitive lipase (HSL), one of the enzymes required to break down triacylglycerides into fatty acids, was used as a marker of lipolysis. Expression of HSL was not significantly increased in cold-acclimated iBAT (Fig. 3a) perhaps due to depletion of easily accessible lipid stores of the multilocular brown adipocytes by 3.5 weeks of cold exposure (Figs. 1 e, f and 2a, b). Increased expression of HSL in cold-acclimated ingWAT (Fig. 4a) is consistent with the need for FAs to power thermogenesis within the tissue and for export as non-esterified fatty acids in the blood for use by iBAT. Significantly decreased HSL in gWAT of cold-acclimated mice (Fig. 5a) was somewhat surprising, though visceral fat is more resistant to induction of lipid mobilization than ingWAT (Wajchenberg 2000). From these data, we conclude that the study design was effective and that mice housed at 4 °C for 3.5 weeks had undergone a significant induction of adaptive thermogenesis displaying a characteristic phenotype of cold-acclimation.

We hypothesized that if PACAP, VIP, or PACAP receptors were important in regulating the thermogenic response to cold stress directly at the adipocyte, their mRNA would be differentially expressed in thermogenic adipose tissues (iBAT and ingWAT), but not non-thermogenic adipose tissues (gWAT) in response to 4 °C housing temperature. PAC1R mRNA was downregulated in iBAT of cold-acclimated mice (Fig. 3a), suggesting PACAP does not participate directly in the maintenance of thermogenetic pathways in iBAT once cold acclimation has been achieved. VPAC1 and VPAC2 receptors and VIP mRNA were not significantly changed in iBAT in response to cold acclimation (Fig. 3b) and thus are also not likely to be important to thermogenic response of brown adipose tissue. In ingWAT, a white adipose tissue depot which “browns” in cold acclimation, PAC1R mRNA was significantly increased in cold-exposed ingWAT, which warrants functional studies assessing PACAP’s ability to activate thermogenesis, promote beige adipocyte recruitment, or mediate transdifferentiation of white to beige adipocytes. In gWAT, PAC1R was downregulated in cold-acclimated mice compared to thermoneutral mice. Evidence presented here shows that PACAP receptors are expressed in adipocytes of brown and white adipose tissues, but of the three PACAP receptors studied, PAC1R is the only receptor differentially expressed in response to cold acclimation and has a depot-specific response to cold-acclimation.

The expression pattern of PAC1R in response to cold acclimation was not as simple as we had hypothesized, with upregulation of the PAC1R in the inducible ingWAT adipose tissue depot but downregulation in iBAT of cold-acclimated mice. The uncoordinated expression of PAC1R and UCP1 does not support a role for PACAP in regulating the expression of this thermogenic protein at the level of the adipocyte. Interestingly, in all three adipose tissues studied, PACR1R and HSL showed similar trends in regulation in response to housing temperature with both being downregulated in iBAT and upregulated in ingWAT in response to cold-acclimation. Instead of directly regulating thermogenic proteins, PAC1R may be affecting lipid and/or glucose metabolism or sensitizing adipocytes to potentiate greater adrenergic receptor response. Previously, PACAP has been shown to have both catabolic and anabolic effects on adipocyte glucose and lipid metabolism in vitro (Akesson et al. 2005; Akesson et al. 2003). When a relatively high concentration (10 nM) of PACAP38 is administered to cultured primary adipocytes from rat, a sixfold increase in lipolysis, as measured by glycerol release, occurs and this response is decreased to a twofold induction of lipolysis at 1 nM PACAP38. Lipolytic function in these studies was associated with induction of PKA activity and is abolished by the simultaneous administration of insulin. Furthermore, administration of PACAP38 and insulin in combination increases lipogenesis by 30% compared to cells receiving insulin alone (Akesson et al. 2003). This finding demonstrates a need for future functional experiments to characterize a lipolytic role for PACAP in different adipose tissues and to better understand PAC1R’s interactions with other important receptors, such as those for insulin and leptin. Given the pleiotropic action of PACAP and the variety of functionally distinct splice variants of the PAC1R, it is possible that PACAP has distinct roles in different adipose tissue depots.

In summary, this work provides foundational expression data to inform future studies aimed at deciphering signal transduction mechanisms and the functional role PACAP performs in different types of adipose tissue, including a potential role for PACAP in the peripheral regulation of energy metabolism.
